# Simple and green technique for sequestration and concentration of silver nanoparticles by polysaccharides immobilized on glass beads in aqueous media

**DOI:** 10.1186/s13065-015-0110-7

**Published:** 2015-06-09

**Authors:** Alaeddine Kibeche, Alexandre Dionne, Roxanne Brion-Roby, Christian Gagnon, Jonathan Gagnon

**Affiliations:** Département de Biologie, chimie et géographie, Université du Québec à Rimouski, 300 allée des Ursulines, Rimouski, QC G5L 3A1 Canada; Centre Saint-Laurent, Environment Canada, 105 McGill st., 7th floor, Montreal, QC H2Y 2E7 Canada

**Keywords:** Silver, Nanoparticles, Supported polymers, Polysaccharides, Chitosan, 2-hydroxyethylcellulose, Glass beads

## Abstract

**Background:**

Engineered nanoparticles have unique properties compared to bulk materials and their commercial uses growing rapidly. They represent a potential risk for environment and health and could be eventually released in water. Silver nanoparticles (Ag NP) are applied in various products and are well-known for their antibacterial properties. Nowadays, pre-concentration and separation methods for Ag NP possess some limitations. Here, we present a simple, green method to sequestrate and concentrate Ag NP from different aqueous media.

**Results:**

Supported polysaccharides on glass beads synthesized in water by a single step reaction show high sequestration capacity of citrate-coated Ag NP in aqueous media. Supported polysaccharides were characterized by infrared spectroscopy, scanning electron microscopy (SEM) and elemental analysis. Sequestration of 83.0 % of Ag NP was attained from a 20 μg.L^−1^ aqueous solution with supported chitosan in water whereas supported 2-hydroxyethylcellulose (HEC) reached 64.0 % in synthetic seawater in 2 h. The influence of polymer/glass beads ratio and molecular weight of polysaccharides was also studied. The effect of the salinity and humic acids on sequestration of Ag NP was investigated. Supported polymers have shown high performance for sequestration of ionic silver. Sequestration of 82.5 % and 80.8 % were obtained from a 60 μg.L^−1^ silver ion (as nitrate salt) with supported HEC and chitosan, respectively. Sequestrated Ag NP was characterized with transmission electron microscopy (TEM) where images showed Ag NP with unchanged size and shape.

**Conclusions:**

This sequestration method, involving green synthesis, allows efficient concentration and characterization of Ag NP from different aqueous media. This simple and fast method is a potential sustainable technique for elimination of Ag NP and ionic silver from waste waters and waters at different salinities.

## Background

Nanomaterials possess different useful properties compared to their bulk materials, therefore their commercial utilization grows rapidly [1]. However, nanomaterials represent a potential risk for the environment and health. Pulmonary, dermal and oral exposures of Ag NP lead to absorption of silver and to inflammatory, genotoxic, and cytotoxic events [2]. Silver nanoparticles (Ag NP) are used in various fields such as medicine, electronics, cosmetics, chemical industry, food technology, and sporting goods. They are well-known for their highly effective antibacterial activity against a broad spectrum of bacterial strains [3, 4]. The Ag NP would eventually be released in water where they have potential adversary effects such as on nitrogen removal in municipal wastewater treatment plants and may limit the disposal of the biosolids as agricultural fertilizer [5, 6]. Ag NP can be removed from aqueous dispersions using adsorption-based methods [5–7]. Some approaches have been reported for the pre-concentration and separation of metal-based NP with some limitations. Liquid-liquid extraction typically involves large amount of organic solvents [8]. Cloud point extraction that can be applied to small volumes [9]. Asymmetric flow field-flow fractionation (AF^4^) coupled to ICP-MS involves costly instrumentation [10–12].

Polysaccharides are used in many fields such as medicine, industry, nutritional product, enzyme immobilization and environment [13]. Cellulose and chitosan are the two most abundant polysaccharides on earth. Chitosan presents a high efficiency for metal ions coordination that is limited by its insolubility in aqueous solutions and organic solvents [14]. However, chitosan can be solubilized in diluted aqueous solutions. Cellulose derivatives like 2-hydroxyethylcellulose (HEC) are widely used in environmental studies due to its solubility in water [15, 16].

Polysaccharides like HEC and particularly chitosan are known for their sorption capacity of metal ions [17, 18]. Indeed, extraction of metals ions by these two polymers is hampered by solubility in different aqueous solutions leading to a need for their immobilization on inorganic supports. Some recent studies have shown the ability to modify the glass beads surface by grafting polysaccharides using organic linkers for coordination of dissolved transition metals [14, 19, 20].

In this study, we present a simple and efficient immobilization of HEC and chitosan directly on glass beads through silanol groups, leading to an insoluble support presenting a high sorption capacity for citrate-coated Ag NP.

## Results and discussion

### Polymer immobilization methods

**Scheme 1:**
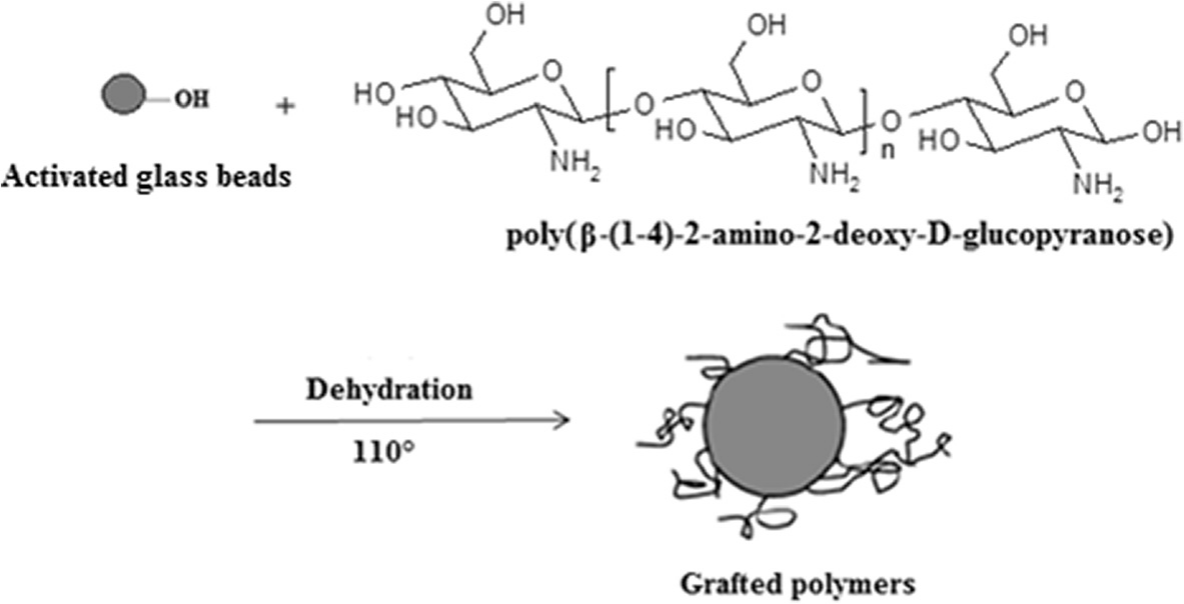
Immobilization of chitosan on glass beads

Immobilization of the polymers 2-hydroxyethylcellulose (HEC) and chitosan on glass beads can be achieved using several reaction steps [14, 20]. Polymers are promising due to the high number of repetitive units and therefore to their large capacity of adsorption on a surface. The adsorption on Ag NP is facilitated by their high surface/mass ratio [3]. As we want to develop a support that is easily synthesized, the direct reaction between glass beads and polysaccharides was attempted; prior the glass beads surface was activated to increase the silanol group concentration. Glass beads and polysaccharides solutions were mixed together and heated over 110 °C to eliminate water molecules, leading to insoluble supports. The silanol groups on glass beads surface would react with alcohol groups of polysaccharides through a dehydration reaction forming Si-O-C bonds. Polymers were immobilized on glass beads surface as illustrated in Scheme 1 offering a high surface similarly to dendrimers.

### Characterization of supported polymers

**Fig. 1:**
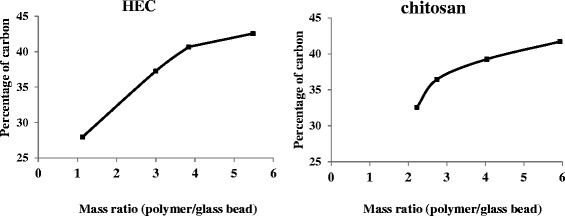
Percentage of carbon in supported polymers with different polymer/glass beads ratios

In the aim to study the influence of polymer/glass beads mass ratios on Ag NP sequestration, some supports have been synthesized with five different ratios of HEC and chitosan. The resulting supports were characterized by IR spectroscopy and elemental analysis to confirm the fixation of those polysaccharides on the glass beads. Results of elemental analyses for different ratios are shown in Fig. 1. The percentage of carbon increases with the quantity of HEC to reach a maximum at a mass ratio of five. For chitosan, the percentage of carbon increases also when the mass ratio increases and does not reach a maximum inside the studied range. These increases in polysaccharides content obtained even after prolonged washing with water or diluted acetic acid show that HEC and chitosan can be immobilized on glass beads by simple heating. This reaction represents a rapid and green methodology for the polysaccharides immobilization on glass beads.

### Infrared spectroscopy characterization

**Fig. 2:**
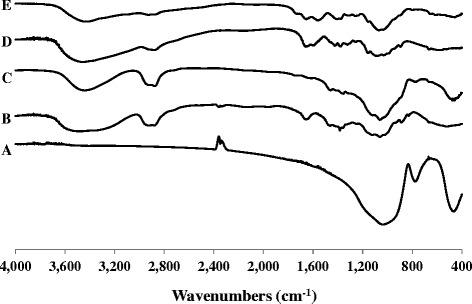
IR spectra of (**a**). Activated glass beads (**b**). HEC, (**c**). Supported HEC, (**d**). Chitosan, (**e**). Supported chitosan

Immobilized polysaccharides were characterized by infrared spectroscopy. An intense IR band at 1100 cm^-1^ assigned to Si-O stretching (Fig. 2) was observed for glass beads whereas HEC shows polysaccharides characteristic bands at 3459 (O-H stretching), 2877 (C-H stretching), and 1000–1150 cm^-1^ (C-O stretching). In addition, IR spectrum of chitosan (Fig. 2) displays bands at 1652 and 1630 cm^-1^ attributable to carbonyl of residual acetyl groups and to NH_2_ deformation, respectively. The major difference between polymers and immobilized polymers was the enhanced relative intensity of 1100 cm^-1^ band probably due to some Si-O absorption, despite the fact that infrared beam can slightly reach the glass beads surface.

### Sequestration of silver nanoparticles with supported chitosan

Citrate-coated Ag NP were chosen because they are widely used in industry and are easily commercially available. The concentration of silver was determined in the supernatant and the support in order to perform a mass balance and confirming the complete recovery of the silver. The results of sequestration by supported chitosan are presented in Table 1. Sequestrations were performed using five polymer/glass bead mass ratios of immobilized high molecular weight chitosan. Around 35 % of silver from a 100 μg.L^−1^ of Ag NP solution was sequestrated by supports of ratios three and five (runs 2 and 4).

**Table 1:** Sequestration of Ag NP with supported chitosan

Run	Polymer/glass bead ratio	Concentration of Ag NP (μg.L^−1^)	Salinity (PSU)^a^	Viscosity (cP)	Percentage of sequestration (%)^b^
Influence of polymer/glass bead ratio					
1	2	100	0	500	26.2
2	3	100	0	500	35.4
3	4	100	0	500	23.2
4	5	100	0	500	34.3
5	6	100	0	500	27.2
Influence of molecular weight and quantity of support used					
6	3	100	0	500	35.4
7	3	100	0	20	48.5
8	5	10	0	500	34.3
9	5	100	0	20	52.7
10	5 with double quantity	100	0	20	63.0
Influence of salinity					
11	5	20	0	20	83.8
12	5	20	35	20	73.6
13	5	40	0	20	58.7
14	5	40	35	20	54.5
15	5	60	0	20	76.6
16	5	60	35	20	64.9

a) PSU: practical salinity unit; b) the relative uncertainty is lower than 8 %

### Influence of chitosan molecular weight

Two ratios (i.e., polymer/support) showing the maximum of sequestration (ratios three and five) were synthesized with low molecular weight chitosan (20 cP) and were used for Ag NP sequestration at a concentration of 100 μg.L^−1^. With a mass ratio of five and using a low molecular weight, a sequestration of 52.7 % was attained (run 9), which was higher than with high molecular weight chitosan (run 4). When twice quantity of supports of ratio five was used (0.6 g instead of 0.3 g), the percentage of sequestration to Ag NP increased from 52.7 (run 9) to 63.0 % (run 10). These results indicate that lower molecular weight and higher quantity of supports lead to an increased percentage of sequestration. A higher quantity of support avoids its saturation and permits to reach a sequestration efficiency that is not limited by the quantity of support under these conditions.

### Sequestration of Ag NP with supported HEC

The results of sequestration with supported 2-hydroxyethylcellulose (HEC) are shown in the Table 2. Like for chitosan, the sequestrations were performed using five mass ratios of high molecular weight HEC (720 kDa) and 100 μg.L^−1^ of Ag NP (runs 17–21). In these conditions, the best sequestration was achieved with the mass ratio of four leading to a percentage of 60.9 % (run 19). On this basis, the subsequent analyses were then realized with this ratio.

**Table 2:** Sequestration of Ag NP with supported HEC

Run	Polymer/glass bead ratio	Concentration of Ag NP (μg.L^−1^)	Salinity (PSU)^a^	Molecular weight (kDa)	Percentage of sequestration (%)^b^
Influence of polymer/glass bead ratio					
17	2	100	0	720	37.0
18	3	100	0	720	51.4
19	4	100	0	720	60.9
20	5	100	0	720	12.4
21	6	100	0	720	39.7
Influence of molecular weight and salinity					
22	4	20	35	720	64.0
23	4	20	35	92	73.8
24	4	60	35	720	31.0
25	4	60	35	92	39.6
26	4	60	0	92	63.6
Influence of Ag NP concentration					
27	4	90	35	92	48.5
28	4	150	35	92	34.4
29	4	260	35	92	23.0
30	4	300	35	92	20.5

a) PSU: practical salinity unit; b) the relative uncertainty is lower than 8 %

### Influence of HEC molecular weight

Sequestrations were also performed by immobilized HEC with high and low molecular weight (720 and 92 kDa) in synthetic seawater (35 PSU). As for chitosan in water (0 PSU), the percentage of sequestration increased with the utilization of lower molecular weight. Indeed, at Ag NP concentration of 20 and 60 μg.L^−1^, the percentage of sequestration increased from 64.0 (run 22) to 73.8 % (run 23) and from 31.0 (run 24) to 39.6 % (run 25), respectively.

### Influence of Ag NP concentration on sequestration with immobilized HEC

Six concentrations of citrate Ag NP (20–300 μg.L^−1^) were prepared and sequestrated by immobilized low molecular weight HEC (92 kDa) in synthetic seawater (35 PSU, runs 23, 25–30). The percentage of sequestration decreased when concentration of Ag NP increased. At 20 μg.L^−1^ percentage of sequestration was 73.8 % (run 23) and decreased to 20.5 % when the concentration increased to 300 μg.L^−1^ (run 30). This phenomenon can be explained by equilibrium between free Ag NP and sequestrated Ag NP by the support that is consistent with the result obtained in run 10. In these conditions (run 30), the support saturates at 2 μg of Ag NP per gram of HEC support.

### Influence of salinity on sequestration

The study of salinity is important in this work because it can influence aggregation of Ag NP and shows the applicability of the sequestration method to samples in more complex media. Such aggregation was reported as stronger in the presence of divalent ions like calcium cations [21]. Also, the presence of chloride ions can create electrostatic bridges with silver and causes the precipitation of Ag NP in silver chloride forms. [22].

Effect of salinity on the Ag NP sequestration was studied using synthetic seawater (35 PSU) that includes all major ions. With supported chitosan (Table 1), percentages of sequestration in water were always superior to those in synthetic seawater. At a concentration of 20 μg.L^−1^, this percentage decreased from 83.8 (run 11) to 73.6 % (run 12). A decrease in sequestration efficiency was also observed at concentrations of 40 and 60 μg.L^−1^, decreasing from 58.7 (run 13) to 54.5 % (run 14) and from 76.6 (run 15) to 64.9 % (run 16), respectively. With supported HEC (Table 2), percentage of sequestration also decreased from 63.6 (run 26) to 39.6 % (run 25) at a concentration of 60 μg.L^−1^ of Ag NP. On this basis, the presence of salts influences negatively the sequestration efficiency. This is probably due to precipitation of Ag NP in silver chloride forms and NP aggregation.

### Influence of humic acids on sequestration

**Fig. 3:**
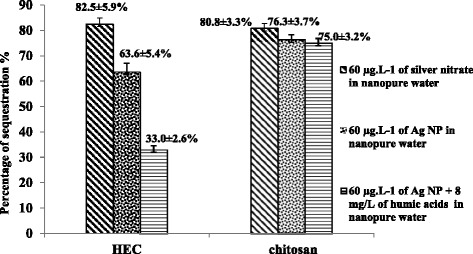
Sequestration of Ag NP and silver nitrate by immobilized polysaccharides

The influence of organic matter was studied by addition of standard humic acids from Suwannee River. Study of organic matter is important in our work because it can influence the processes of adsorption between Ag NP and immobilized polymers. Moreover, the humic acids have been reported to be able to reduce ionic silver and to help independent transformations like aggregation [23]. The sequestration was performed at a concentration of 60 μg.L^−1^ of Ag NP in water containing 8 mg.L^−1^ of humic acids. Results in Fig. 3 show that the percentage of sequestration decreases when humic acids are added. The decrease was more significant with supported HEC passing from 63.6 to 33.0 %. In comparison, the percentage of sequestration with supported chitosan was kept almost identical from 76.0 to 75.0 %. Thus, the addition of humic acids affects the sequestration of Ag NP by a possible sorption or interaction with immobilized polysaccharides.

### Sequestration method selectivity

In the environment, silver is present as many forms like ionic silver and Ag NP. The sequestrations of silver ion (as nitrate salt) realized in duplicate at a concentration of 60 μg.L^−1^ indicated that our supports sequestrated 82.5 ± 5.9 % and 80.8 ± 3.3 % of Ag(I) with immobilized low molecular weight chitosan and HEC, respectively. These results of sequestration show that supported polymers are not selective and are able to sequestrate both Ag NP and silver ions.

### Electron microscopy-based characterization of sequestrated Ag NP

Several studies have presented techniques of characterization of Ag NP but it is always difficult to distinguish between different speciation of Ag NP [24–26]. Most popular tools for characterization of Ag NP are transmission electron microscopy (TEM) and scanning electron microscopy (SEM). TEM is the most sensitive tool for characterization of Ag NP where properties such as the state of aggregation, dispersion, sorption, size, structure and shape can be observed [27].

### Transmission electron microscopy

**Fig. 4:**
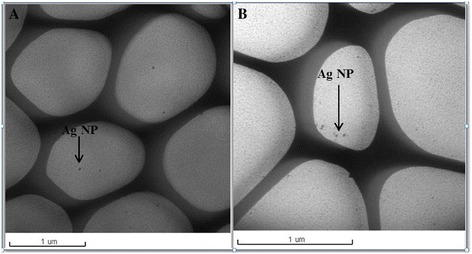
TEM images of sequestrated Ag NP with supported polymers, Ag NP from supported chitosan (**a**), Ag NP from supported HEC (**b**)

The electron beam in TEM is transmitted through in crossing thin sample and has interacted with the particles when the non-absorbed electrons are focused onto an imaging detector [28]. This technique allows identifying distribution and size of Ag NP. Ag NP sequestrated by supported polymers were observed in TEM (Fig. 4). The average size and shape of Ag NP appeared still unchanged at 20 nm after sequestration. However polymers cannot be observed by this technique because they are composed of only light elements. Organic matter would need to be stained by a heavy metal cocktail in order to be visible and imaging of lighter atoms in an electron microscope is more difficult as they scatter electrons less efficiently [27, 28].

### Scanning electron microscopy

**Fig. 5:**
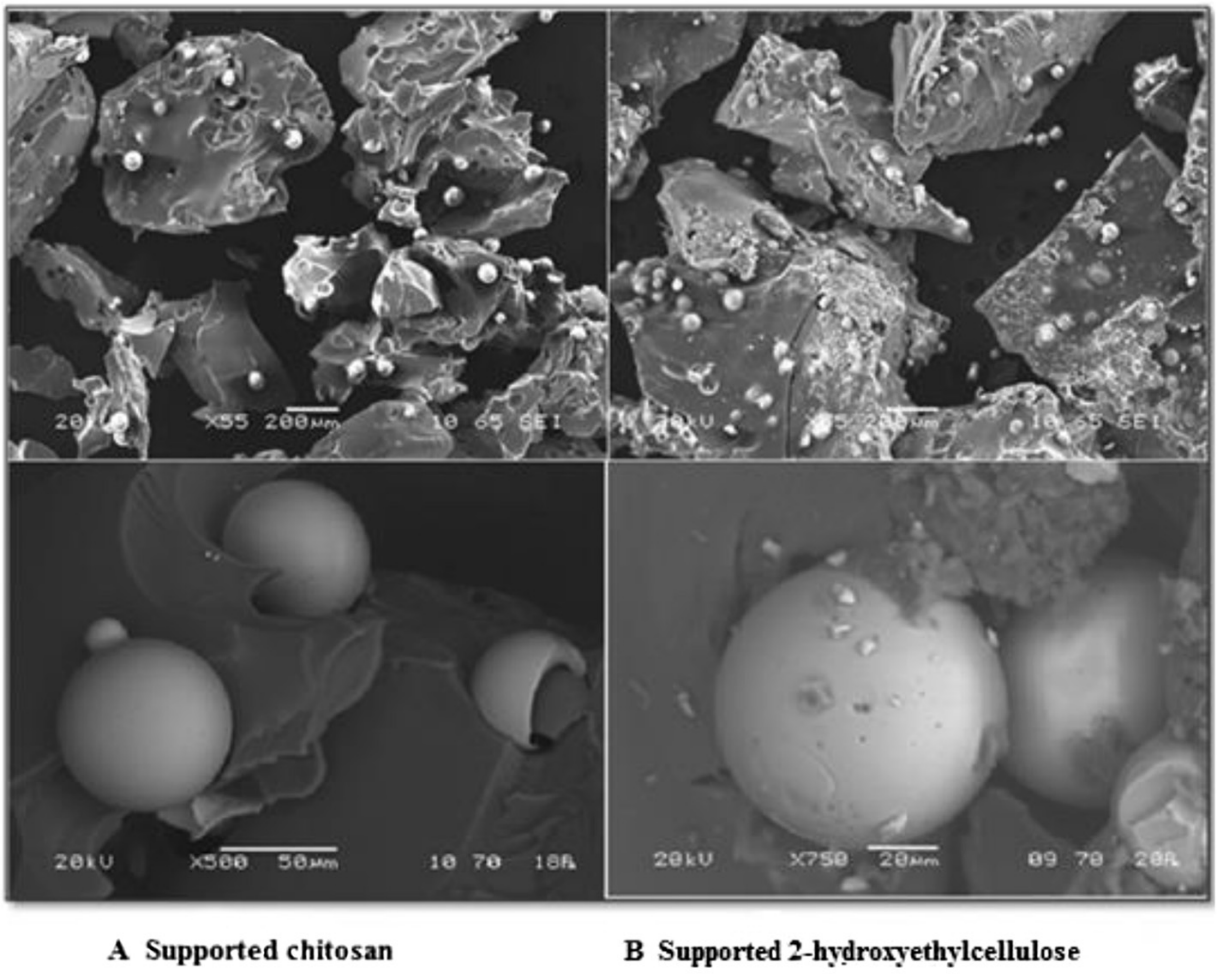
SEM images of sequestrated Ag NP with supported polymers, Supported chitosan (**a**), Supported HEC (**b**)

In SEM, the sample is exposed to a high energy electron beam and the interaction of the beam with the particle surface are scanned over the sample and measured as secondary electrons [28]. Figure 5 represents a SEM image of Ag NP sequestrated by supported polymers when the glass beads are fixed in polymeric matrices. In the other hand, it was impossible to quantify sequestrated silver because the limit of detection of energy-dispersive X-ray spectroscopy for silver is superior to the concentration used. The SEM images show that glass beads and polymers are fixed together in a polymeric matrix in the solid state.

## Experimental

### General information

Glass beads (<106 μm), HEC with two molecular weights (90 and 720 kDa), humic acids of Suwannee River, ultrapure grade of nitric acid (≥70 %) and hydrogen peroxide (≥30 %) were purchased from Aldrich. Chitosans from Nordic shrimps with deacetylation degrees of > 95 % and a viscosity of 20 and 500 cP (1 % in acetic acid 5 % v/v solution) were bought from Primex (Iceland) and Marinard Biotech (Quebec, Canada). Infrared absorption spectra were recorded on a FTIR iS10 spectrophotometer in KBr pellets. Elemental analyses were performed by combustion with costech 4010 analyzer. Citrate coated Ag NP of diameter of 20 nm was obtained from Ted Pella at a concentration of 0.02 mg/mL. Nanopure water was obtained using a nanopure Diamond system (model D11931) from Barnstead. Synthetic seawater was synthesized as reported in literature [29]. Silver was quantified by inductively coupled plasma mass spectrometry (ICP-MS) model Agilent 7500 (Octopole reaction system). Microscopic characterizations were performed by TEM and SEM using respectively on Delong Instruments model LVEM5 and JEOL 646OLV microscopes. In SEM, low magnification images (×55 magnification) were acquired in secondary electrons mode. In order to reduce charging, higher magnifications images were acquired under low vacuum (18–20 Pa) in backscattered electrons mode. For analyses in TEM, the samples were ground and then triturated with dried ethanol. A drop of the ethanolic solutions was placed on 400 mesh copper grid coated with carbon from Ted Pella. The solution was evaporated at rt before to be analyzed.

### Activation of glass beads

Glass beads were activated as described in the literature [14]. Briefly, glass beads (10 mg) in nanopure water (50 mL) were stirred with aqueous 1 M sodium hydroxide solution during 50 min at 50 °C. The suspension was filtered under gravity and washed with water until the filtrate reach pH 7. Glass beads were dried in oven during 12 h at 110 °C prior uses.

### Immobilization of HEC and chitosan

Activated glass beads were added to HEC in nanopure water (50 mL) or to chitosan dissolved in acetic acid solution (5 % v/v, 50 mL) according to the desired polymer/glass beads ratio. The suspension was stirred and heated at 70 °C during 20 min. The suspension was evaporated to dryness with rotary evaporator. The solid was heated in oven during 5 h at 110 °C. Finally, the solid was ground to a fine powder ≤ 2 mm and washed with water (50 mL) for HEC and with acetic acid (5 % v/v, 50 mL) for chitosan to eliminate excess of polymers. The resulting solid was filtered on filter paper and dried at 110°C during 5 h.

### Sequestration of citrate Ag NP

Ag NP solutions were prepared by dilution of the commercial solution using nanopure water. In 15 mL Felkin tubes, 0.3 and 0.6 g of supports of HEC and chitosan, respectively, were added to a solution of Ag NP (10 mL) at a well-defined concentration and stirred with magnetic stirring bar for 2 h. The samples were then centrifuged for 5 min at 1000xg. The supernatant was first recovered with a micropipette and transferred in the tubes and the volume was determined. Thereafter, the supports were recovered by vacuum filtration. Silver concentrations and percentage of sequestration were determined from supernatant and the supports by ICP-MS.

### ICP-MS analyses

Supernatant (2 mL) and all supports after sequestration were separated, then digested individually with ultrapure nitric acid (4 mL) and hydrogen peroxide (1 mL). The tubes were left open for 2 h and heated with a hot bath for an additional period of 2 h at 70 °C. Finally the samples were diluted with water so that the concentration of acid does not exceed 20 %. The solutions were analyzed by ICP-MS using an Agilent ICP-MS 7500C with ChemStation v.3.04 software. Each measure is a mean of 5 replicates; the acquisition time is 0.38 s for a total time of 1.9 s for a measurement. The calibration was made from silver in 2 % HNO_3_ (1.000 mg/L) purchased from Certiprep. The calibration range had 9 points from 0.75 to 200 μg.L^−1^) with a blank (0 μg.L^−1^). The detection limit was 0.01 ng/mL and the quantification limit was 0.05 ng/mL.

## Conclusions

The synthesis of polymeric supports is simple, green and fast. Synthesized supports are insoluble in various aqueous media including synthetic seawater. Supported polymers present high performance for sequestration of Ag NP in aqueous solutions within only 2 h. The results obtained show that sequestration of Ag NP is efficient with more than 83 % of sequestrated Ag NP in water with supported chitosan and 64 % of Ag NP with supported HEC in synthetic seawater from a concentration of 20 μg.L^−1^.

The presence of salts and humic acids decreases the sequestration of Ag NP. The humic substances influence the sorption between supported polymers and Ag NP probably by adsorption. Percentage of sequestration in water is superior comparatively to synthetic seawater (35 PSU), around 10 % for chitosan and nearly double for HEC supports. Supported polymers also present good performance for sequestration of ionic silver (Ag^+^) with more than 80 % of silver ion removed from a concentration of 60 μg.L^−1^ by supported HEC and chitosan. Images of sequestrated Ag NP obtained by TEM showed distribution, size, and form of sequestered Ag NP.

This method is a potential sustainable technique for elimination of both Ag NP and ionic Ag from waste waters and waters of different salinities. In addition, sequestration of Ag NP allowed their concentration and characterization from aqueous media. It would be interesting to test our method on various environmental samples containing Ag NP species.
